# Medical Uses of Electricity

**Published:** 1867-11

**Authors:** 


Medical Uses of Electricity, with special reference to General Electrization as a Tonic in Neuralgia, Rheumatism, Dyspepsia, Chorea, Paralysis, and other affections associated with general debility. With illustrative cases. By GEO. M. BEARD, M.D., and A. D. ROCKWELL, M.D. New York: WM. WOOD & Co., 61 Walker Street. 1867.

This is a very neatly printed and bound duodecimo volume of 65 pages. The principal part of the matter was originally contained in a series of articles published in the *New York Medical Record;* but both authors and publishers have conferred a favor on the profession by giving it a more permanent and generally accessible form. The chief object of the authors is, to illustrate the *superior efficacy of general electrization* over the more common practice of applying it locally. Practitioners will find this little monograph worthy of a careful perusal.

Price.—Full-bound, $1.00; in paper, $0.75.

